# Interconnected Codons: Unravelling the Epigenetic Significance of Flanking Sequences in CpG Dyads

**DOI:** 10.1007/s00239-024-10172-1

**Published:** 2024-04-18

**Authors:** Leo Douglas Creasey, Eran Tauber

**Affiliations:** Department of Evolutionary and Environmental Biology, and Institute of Evolution, University of Haifa, 199 Abba-Hushi Avenue, Haifa, 3498838 Israel

**Keywords:** DNA methylation, Epigenetics, Coding DNA, Codon usage bias

## Abstract

Hypothesizing that CpG codon dyads, formed by consecutive codons containing a cytosine-guanine pair (NNC-GNN), may play a crucial role in gene function, we conducted an extensive analysis to investigate their distribution and conservation within mammalian genes. Our findings reveal that genes characterized by a high density of CpG codon dyads are notably associated with homeobox domains and RNA polymerase II transcription factors. Conversely, genes exhibiting low CpG codon dyad density have links to DNA damage repair and mitosis. Importantly, our study identifies a re-markable increase in expressed genes that harbor CpG during embryonic development, suggesting their potential involvement in gene regulation at these developmental stages. These results under-score the functional significance of CpG codon dyads in DNA methylation and gene expression, fur-ther demonstrating the coevolution of consecutive codons and their contribution to codon usage bias.

**Supplementary Information:**

The online version contains supplementary material available at 10.1007/s00239-024-10172-1.

## Introduction

DNA methylation is a major epigenetic marker that usually takes the form of 5-methylcytosine (5mC). In the human genome 5mC is pervasive (70–80%) and plays important roles in a variety of cellular processes, including retrotransposon silencing, genomic imprinting, X-chromosome inactivation, regulation of gene expression, and maintenance of epigenetic memory (Greenberg and Bourc’his 2019). Importantly DNA methylation has been also implicated in cancer development and aging and is often associated with alterations in methylation patterns which can inhibit the expression of essential genes or facilitate the abnormal expression of detrimental genes (Sproul and Meehan 2013).

In metazoan, DNA methylation is present in the context of CpG dinucleotides (Zemach et al. 2010), which is due to the high specificity of the DNA methyltransferase 1 (Dnmt1). The methyl group attached to the cytosine can lead to spontaneous deamination, leading to a C -> T mutation. Consequently, animals that utilize DNA methylation tend to have a 5 times reduced level of CpG’s relative to what would usually be expected (Sved and Bird 1990). Clusters of CpG sites called CpG islands, which are located in promoter regions, are often found unmethylated. Methylation of CpG island impedes transcription, and the extent of inhibition increases with the density of CpG dinucleotides at the promoter regions (Weber et al. 2007).

Along with the research interest in promoter regions, it was found that substantial DNA methylation also takes place in gene bodies, and is particularly abundant in genes that show intermediate levels of expression (Zemach et al. 2010). In gene body regions, exons tend to be more methylated than introns, which suggests that DNA methylation in gene bodies has a role in the elongation and termination of transcription as well as potentially influencing splicing (Brenet et al. 2011; Shayevitch et al. 2018).

In a landmark paper (Branciamore et al. 2010), DNA methylation in the coding region was analyzed by studying the distribution of synonymous amino-acid codons that harbor CpG (e.g. Proline CCG, or Alanine GCG). Their analysis revealed that while most coding regions showed the expected depletion of CpG, around a tenth of protein-coding genes were relatively CpG-rich. Importantly, analysis of these regions in organisms that lack DNA methylation such as *Drosophila melanogaster* and *Caenorhabditis elegans*, did not reveal a similar codon preference, suggesting that DNA methylation of codon’s CpG is functionally important.

It was previously noted (Branciamore et al. 2010), that CpG dinucleotides can also be formed by codon dyads (i.e. NNC-GNN). Analysis of these dyads (referred to as ‘silent CpG’ by Branciamore et al. 2010) revealed an intriguing excess of NCC-GNN↔NCG-GNN transversions in Hox coding regions that was interpreted as a selection for preservation of CpG in these genes. Yet, the frequency of CpG codon dyads across the genome has not been analyzed to date.

**Fig. 1:**
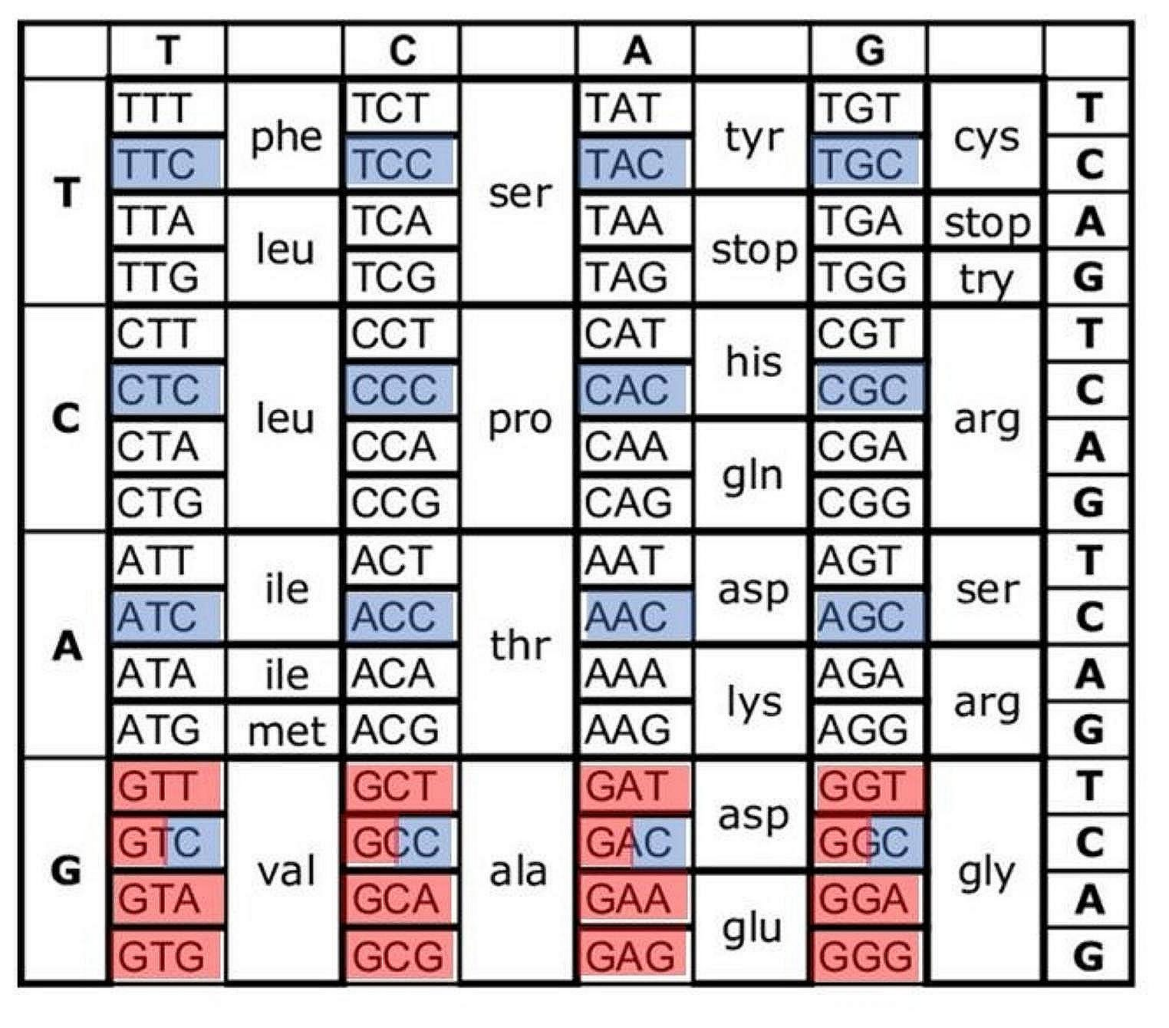
The genetic code. A CpG codon dyad is formed by a codon terminated with a cytosine (colored in blue), followed by a codon starting with a guanine (shaded in red). Four codons can serve as both the first and second codon of a dyad (red-blue color)

The genetic code consists of 12 codons ending with a cytosine, 12 starting with a guanine, and 4 that have ‘G’ and ‘C’ at the first and third positions (Fig. 1). In the current study, we have analyzed CpG codon dyads in a large set of vertebrate ortholog sequences and tested their distribution. We identified conserved dyads in specific genes that may represent selection for epigenetic regulation of gene expression, and adds a new determinant that shapes codon usage bias (CUB).

## Methods

A custom Python script calculated the number of CpG codon dyads each species has in each gene, and the position of each CpG codon dyad, which allowed estimation of the CpG codon dyad conservation across species. CpG codon dyads’ counts per gene, were normalized by its length yielding a density score (relative number of dyads in each gene). For each gene, the overall density was calculated by dividing the number of CpG codon dyads by the consensus size. A CpG codon dyad location was considered to be conserved if ≥ 200 of the 261 species had a CpG codon dyad at that position (76% of species).

The sequence data was acquired from (Bowman et al. 2023). The data included alignments of 13,491 human CDS genes with orthologs from ≥ 250 eutherian species. A phylogenetic tree was also obtained from this study to observe if CpG codon dyad methylation exhibit a phylogenetic signal.

To evaluate functional enrichment among genes with the highest and lowest numbers of dyads, we employed the DAVID bioinformatics server (Dennis et al., 2003). We utilized DAVID v.2021 and considered the entire human gene set as the reference background. Within DAVID, we examined lists and charts containing enriched annotations in the following categories: (1) Biological processes, cellular components, and molecular functions based on Gene Ontology (GO) terms. (2) UP (UniProt) keywords. (3) KEGG pathways. (4) Reactome pathways. (5) Disease annotations.

To test for whether CpG codon dyad occurrence is mirroring gene conservation, we utilized the Phylogenetic Analysis with Space/Time models (PHAST) software package (Hubisz et al. 2011). Using the phyloP tool, we calculated conservation and acceleration scores for each gene. We used the difference between the null and posterior distribution scores and modeled these scores against the number of CpG dyads per 1000 nucleotides.

Bisulfite sequencing data was acquired through the ENCODE online portal (Luo et al. 2020), with the following identifiers: ENCSR464TTP, ENCSR606SSE, ENCSR068MRQ, ENCSR191PVZ, ENCSR082SFX, ENCSR592QDE, ENCSR669BAL, ENCSR493GDU, ENCSR301HQS.

The R software was used for statistical analysis (R Core Team 2023). Phylogenetic analysis was carried out using the R packages “ape” (Paradis and Schliep 2019), “phylobase” (Hackathon et al. 2024) and “phylotools” (Zhang 2017).

## Results

### Functional annotation of genes enriched or depleted by CpG codon dyads

Codon dyads were analyzed in 13,491 protein coding genes from 261 eutherian mammal species. We conducted gene set enrichment analysis of the top 500 genes with the highest density of dyad CpG (average over species). The results show that HOX genes and homeobox domains were highly prevalent in this group. Another prevalent group is a group regulating the binding of RNA POL II (Table 1).

**Table 1:** Go Functional Annotation of Genes Enriched by CpG Codon Dyads

Cluster	ES	Category	Associated term	p-value	Genes (#)
1	45.37	INTERPRO	Homeobox site	3.00E-51	62
		UP_KW	Homeobox	5.65E-48	65
		SMART	HOX	1.87E-43	65
2	35.07	GOTERM_MF_DIR	RNA polymerase II transcription factor activity	1.42E-46	130
		GOTERM_BP_DIR	regulation of transcription from RNA polymerase II promoter	2.55E-38	135
		UP_KW	DNA-binding	1.68E-22	136
3	9.77	SMART	HLH	7.04E-11	21
		GOTERM_MF_DIR	protein dimerization activity	1.11E-06	18
4	7.88	SMART	FH	1.79E-07	12
5	6.5	SMART	BTB	3.86E-06	19

ES = Enrichment score produced by Functional Annotation Clustering in DAVID. Category Terms Defined: UP_KW = Uniprot Keywords; GOTERM MF DIR = GO Term for Direct Involvement in Molecular Function; GOTERM BP_DIR = GO Term for Direct Involvement in Biological Process; SMART protein domain database

When the analysis was extended to encompass the 500 genes with the lowest density, a less pronounced outcome was observed. Nonetheless, notable genes associated with fundamental processes such as DNA repair, mitosis, and centromere functionality were identified (refer to Table 2).

**Table 2:** GO Functional annotation of genes depleted by CpG codon dyads

Cluster	ES	Category	Associated term	p-value	Genes (#)
1	3.43	GOTERM_BP_DIR	cell division	1.20E-04	22
		UP_KW	Mitosis	5.02E-04	18
		UP_KW	Cell division	8.63E-04	22
2	1.99	SMART	PHD	0.01317	7
3	1.94	SMART	PI3Kc	0.00525	4
		GOTERM_MF_DIR	1-phosphatidylinositol-3-kinase activity	0.02068	3
		GOTERM_BP_DIR	phosphatidylinositol-3-phosphate biosynthetic process	0.07684	3
4	1.87	SMART	RRM	0.00744	12
5	1.81	SMART	HELICc	0.00965	8
		SMART	DEXDc	0.01108	8

Our analysis of codon dyads echoes the findings of Branciamore et al. (2010), who highlighted a notable prevalence of CpG single codons within genes from the Hox gene family. This alignment is bolstered by a significant correlation we identified between the abundance of single codon CpGs and CpG-containing codon dyads, illustrated in Fig. S1. This correlation strengthens the concept of a cohesive relationship between CpG-rich codons and their dyadic counterparts, aligning closely with prior observations and underscoring the importance of the presence of CpG sites in these coding genes.

### Specific Sites of CpG Codon Dyads are Highly Conserved

Compared to CpGs found within individual codons, CpG codon dyads are much more prone to coding redundancy. Further, deamination in mammals is expected to increase the selection against the presence of such occurrences (Sved and Bird 1990). Therefore, the presence of CpG codon dyads that are conserved may indicate that they are likely to have a functional purpose.

Nevertheless, analysis of conserved CpG codon dyads (dyadC, where the CpG is present in over 75% of the species) revealed that many genes harbored at least one site that was conserved: 8,328 of 13,491 (62%) had one or more dyadC. The frequency distribution is presented in Fig. S2. The ubiquity of these CpGs across a broad range of mammalian species alludes to a functional role of DNA methylation and the role of natural selection.

For instance, as an arbitrarily chosen example, in the *Tetraspanin*-6 gene (*TSPAN6*), 219 species exhibit a CpG codon dyad at the 20th codon position, formed primarily by a serine-valine dyad (only one other instance of asparagine-valine). Valine, with its four possible codons, accommodates a CpG codon dyad in all cases. Conversely, among the six synonymous codons of serine, only two generate a CpG codon dyad. If there were no selection pressure on CpGs, roughly one-third of sequences would feature a CpG codon dyad. However, codons are unevenly favored. Analyzing coding sequences in the entire data set revealed the actual frequency of redundant codons, with approximately 21.2% of serine codons allowing for a CpG codon dyad. With this true value, we predicted around 55 sequences with a conserved CpG codon dyad out of 261 orthologs. Surprisingly, 219 species indeed conserved this CpG codon dyad, surpassing the prediction by 164 sequences (z-test for proportion, *p* < 0.0001). This example of *TSPAN6*, described above, serves as a randomly chosen representative illustration of the vast majority of dyadC cases.

### Distribution of CpG codon dyads within coding sequences

**Fig. 2:**
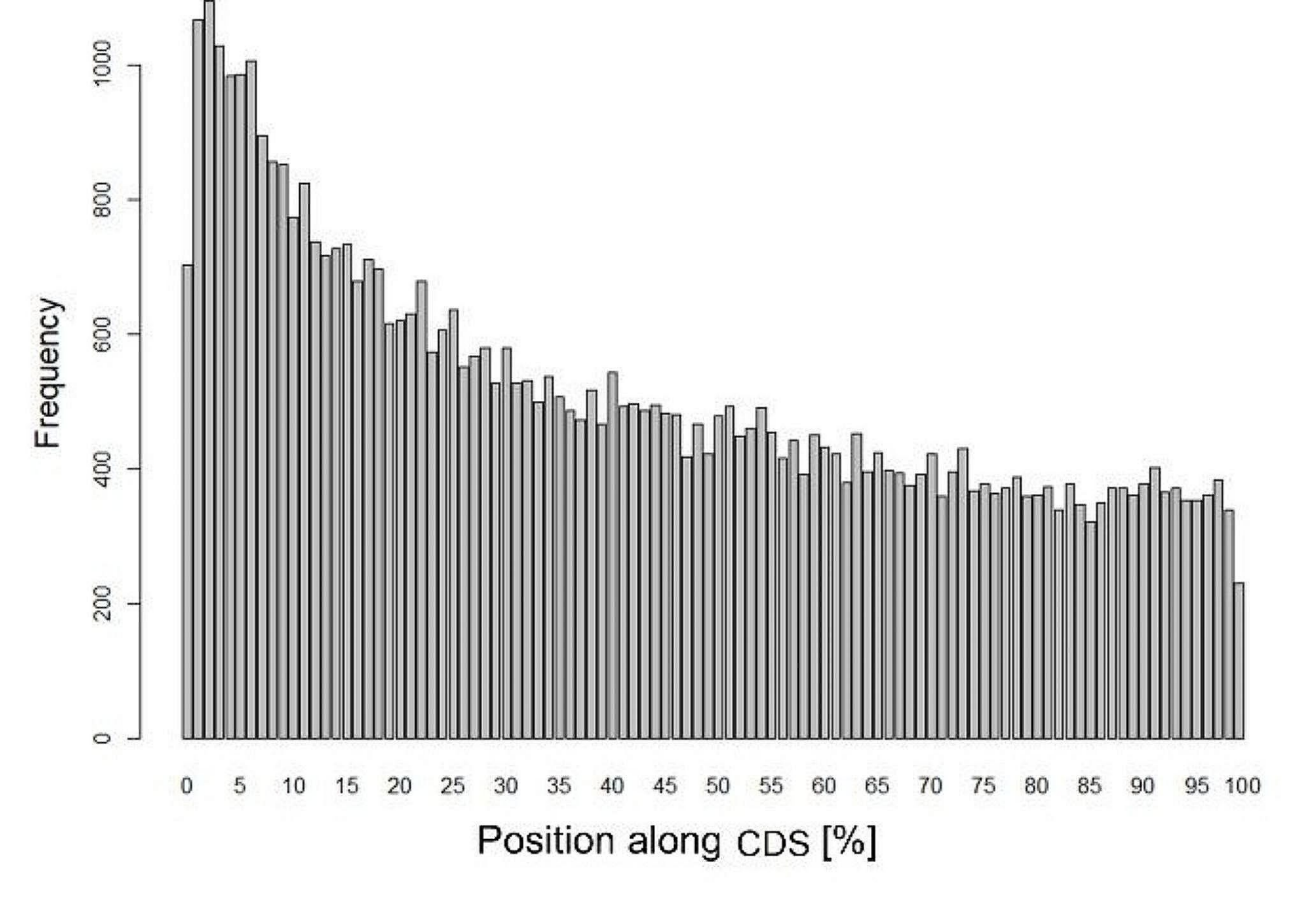
CpG codon dyad frequency along the relative length of the coding sequence (CDS). Analysis is based on 13,491 protein-coding genes from 261 mammalian species. The y-axis represents the frequency of CpG codon dyads

Given the presence of highly conserved locations, we further investigated whether there was any discernible pattern in their distribution across the coding sequence (CDS). The frequency distribution of CpG codon dyads followed an exponential decay, peaking at the beginning of the sequences within the first quarter of the gene (Fig. 2).

We have also tested whether the pattern found in Fig. 2 was equally shared by coding sequences with higher or lower numbers of conserved CpG codon dyads. To that end, we used the top and bottom 1000 CDS (that had at least 1 conserved CpG codon dyad). The frequency distribution of the two groups differs substantially (Fig. S3A-B). Genes with a clear selection away from CpG codon dyads still have conserved dyads near the start of the CDS, suggesting that they must serve a key function in that region. CDS enriched with conserved dyads seem to have these dyads throughout, indicating they may function in some other way. Interestingly, we found the same spatial pattern exhibited by single codon CpG (Fig. S3, C-D). Previous studies showed that CpG islands (CGI) have a preference to overlap with exons, and that synonymous substitution rates of CpG codons are reduced (Medvedeva et al. 2014). We have analysed 13,491 human genes and found a significant correlation between the density of CpG codon dyads and CpG Island (CGI) gene coverage in 13,491 human genes. Spearman correlation analysis demonstrates a positive correlation (ρ = 0.73, S = 2.5e + 10, *p* < 2.2e-16) between the density of CpG codon dyads and the frequency of single codon CpG (Fig. S4).

**Fig. 3:**
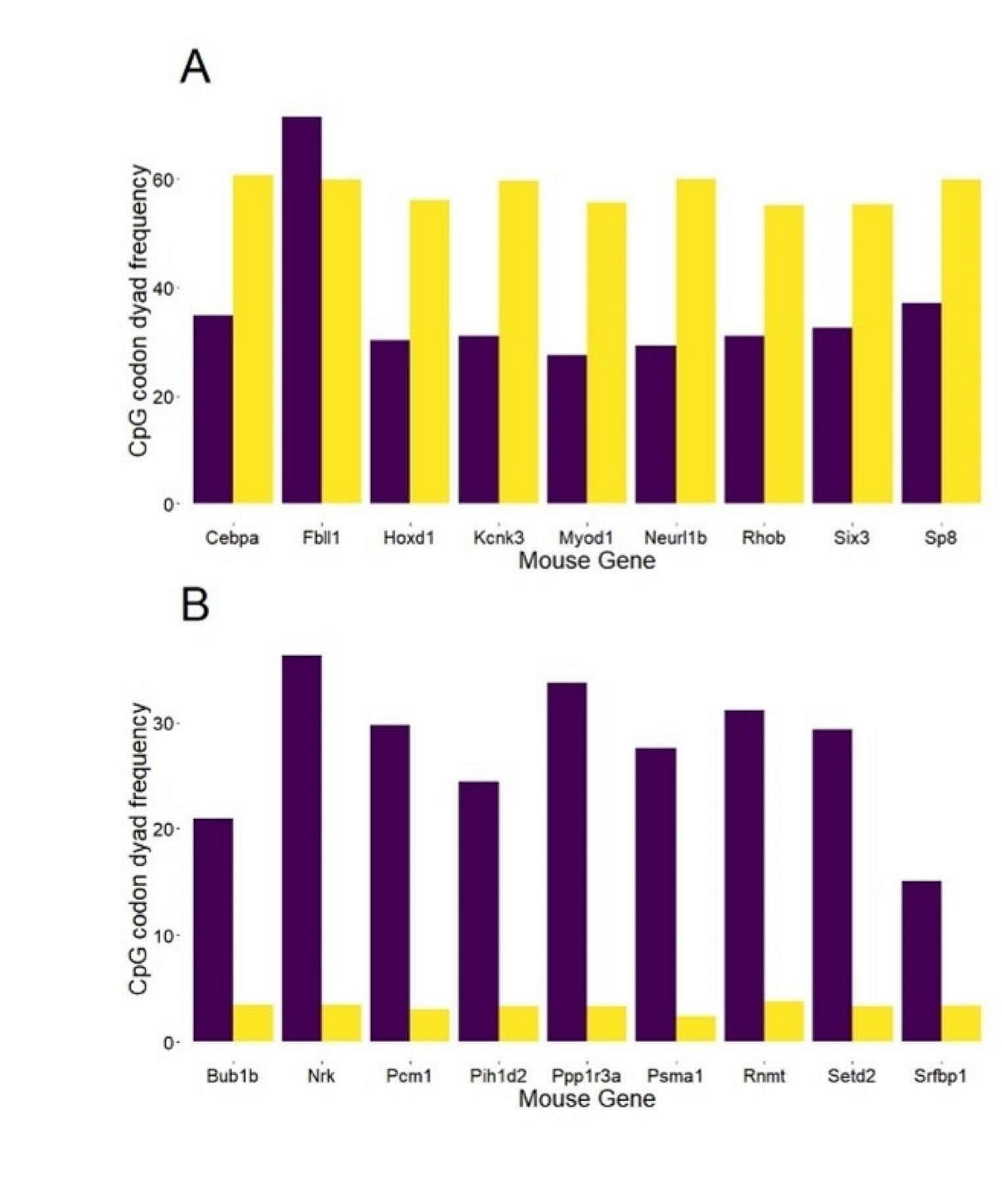
Comparative analysis of CpG codon dyads in homologous genes between *Drosophila* and Mammals. The abundance of CpG codon dyads in *Drosophila* (depicted in purple) and an average value from the mammalian dataset (depicted in yellow) across homologous genes. In panel (**A**), genes exhibiting an enrichment of CpG codon dyads are highlighted, while panel (**B**) focuses on a subset of genes with minimal CpG codon dyad occurrences

Fig. 3 depicts a subset of genes exhibiting a heightened abundance of CpG codon dyads in mammals. Remarkably, across all instances, the prevalence of CpG codon dyads is consistently higher in mammalian genes compared to their *Drosophila* counterparts, underscoring a statistically significant enrichment (Wilcox signed paired test, V = 0, *p* = 1.9e-06). Conversely, when comparing mammalian genes that exhibit a reduced number of CpG codon dyads compared to their *Drosophila* orthologs, the opposite trend was observed (Fig. 3B). Strikingly, in such instances, the number of CpG codon dyads in *Drosophila* genes is significantly higher than in their mammalian counterparts (Wilcox signed paired test, V = 55, *p* = 0.002). Collectively, these findings suggest that the variation in CpG codon dyad occurrences is likely influenced by selection associated with DNA methylation.

Since the conservation of CpG codon dyads may merely be due to conservation of the respective genes, we calculated conservation scores for each gene and tested their impact on CpG dyad abundance (Fig. S5). We found that although there was a significant effect, its magnitude was extremely low (linear model, *p* < 2.2e-16, r^2^ = 0.007). Overall, this suggests that CpG codon dyads are linked to general gene conservation, but this association only explains a small amount of the variation.

To test whether the CpG’s codons (both single and dyads) that we identified are being actually methylated, we analyzed the ENCODE epigenomes datasets from four human individuals (Rozowsky et al. 2023), alongside whole genome bisulfite sequencing data from the HeLa cancer cell line (Dunham et al. 2012).

**Fig. 4:**
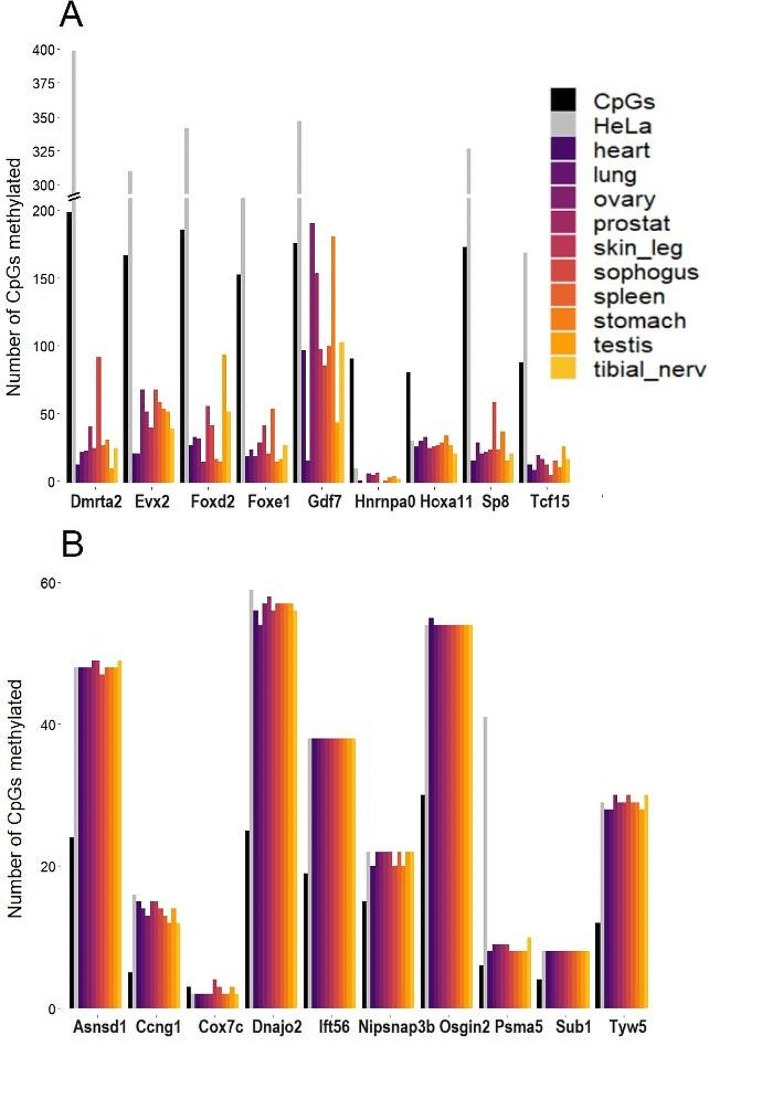
DNA methylation of CpG sites in CDS in different human tissues and HeLa cells. The number of methylated sites in genes with a large number of CpG codon dyads (**A**) and in genes with a low number of CpG codon dyads (**B**) is depicted. The number of CpG sites in each gene’s CDS is indicated (black columns), as well as methylated sites in HeLa cells (grey). Note: The Y-axis scales in panels A and B are different

Fig. 4 shows the results for genes with either a high or low number of CpG codon dyads (top 1000 vs. bottom 1000 genes). In genes with a high proportion of CpG codon dyads (Fig. 4A), many CpG sites are unmethylated. For instance, the *Foxd2* gene has 187 CpG sites in its coding sequence, but on average, only 39.3 are methylated across the 10 organs tested (21%). In contrast, for genes with a low number of CpG codon dyads (Fig. 4B), 9 out of the 10 genes tested show complete symmetric methylation of all available CpG sites across all organs. The HeLa cancer cell line exhibits, as one may expect, hypermethylation in both types of genes (Fig. 4A, B). In low dyad number genes (Fig. 4B) all CpG sites are symmetrically methylated on both strands, a characteristic of its cancerous nature.

### Evolution of CpG Codon Dyads Across Mammalian Phylogeny

**Fig. 5:**
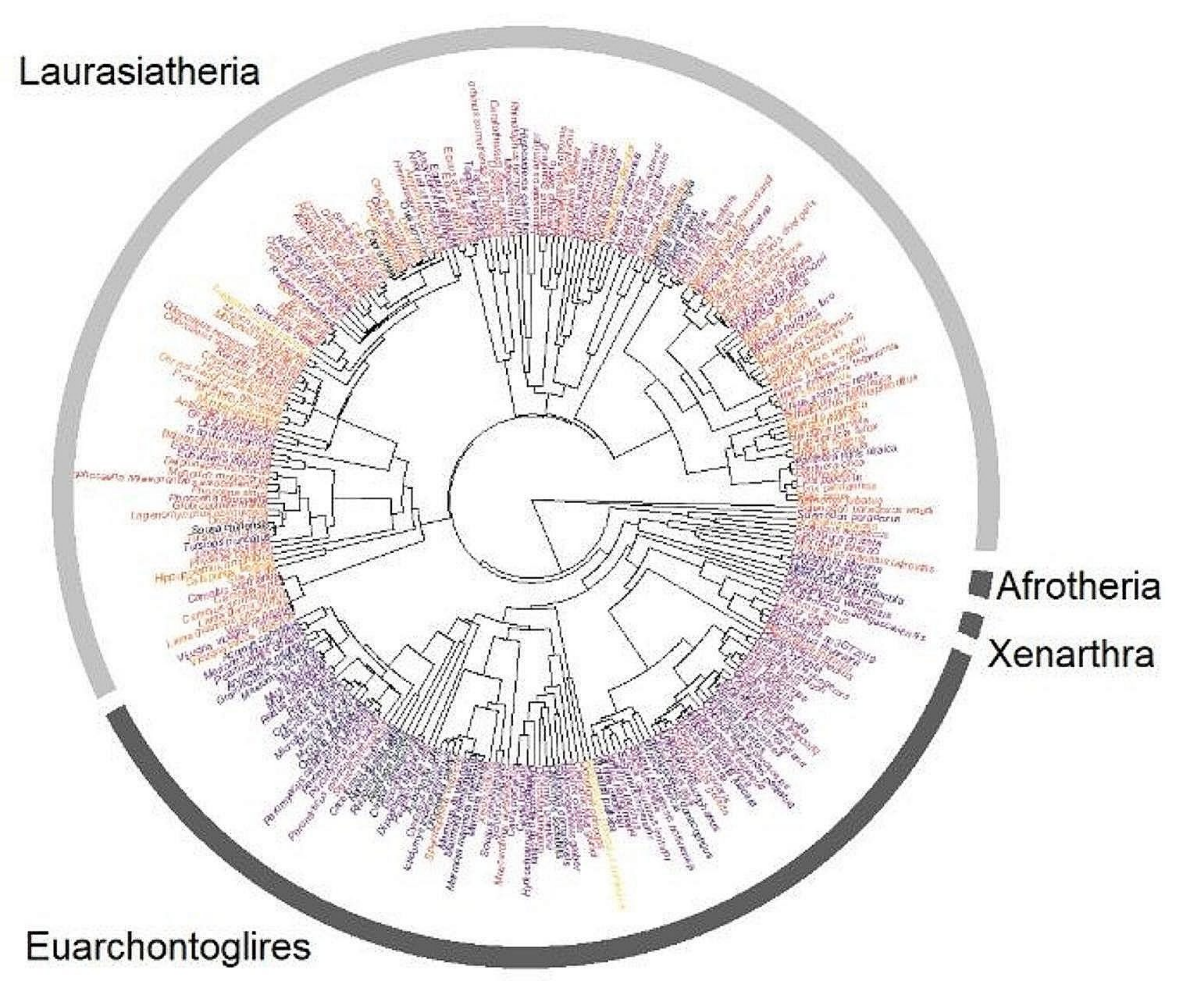
The evolution of the GpG codon dyads across the eutherian mammalian phylogeny. The abundance of CpG codon dyads in 261 mammalian genomic CDS is shown. The colors of the species labels represent the total number of CpG dyads (black-purple: 250–350 × 10^3^, orange-yellow 450–550 × 10^3^). Readers are advised that the labels are not intended for direct species identification but rather for representing dyad abundance. The tree is adapted from (Bowman et al. 2023)

The wide range of eutherian mammalian orders included in this study provided a unique opportunity to test the phylogenetic signal of CpG codon dyad frequency. We mapped the total genomic number of CpG dyads onto the tips of the mammalian phylogenetic tree from Bowman et al. (2023), as shown in Fig. 5.

Graphical exploration of the data, as well as comparisons across different taxonomic orders (Fig.S6), indicate that the CpG dyad count may be influenced by the evolutionary relationships among mammalian orders. Orders that are more closely related and share a more recent common ancestor (e.g., within the Laurasiatheria clade) tend to have similar CpG counts, while more distantly related orders (e.g., between Laurasiatheria and Euarchontoglires) exhibit more divergent CpG dyad counts. Higher CpG dyad counts were higher in Laurasiatheria compared to Euarchontoglires (Fig. 5).

We used Pagel’s λ to test for phylogenetic signal (Münkemüller et al. 2012). The results indicated a significant phylogenetic signal (λ = 0.79, *p* = 1.2e-40). However, when the Blomberg’s K test was applied, the null hypothesis was not rejected (K = 0.026, *p* = 0.35). The discrepancy between the two tests, where the null hypothesis was rejected by Pagel’s lambda but not by Blomberg’s K, may indicate that the CpG dyad counts exhibit a phylogenetic signal, but the pattern of trait evolution deviates from strict Brownian motion expectations, which both tests assume. This could be due to factors such as stabilizing selection, adaptive radiation, or other evolutionary processes that cause the trait to deviate from Brownian motion (Münkemüller et al. 2012).

### Abundance of CpG Codon Dyads is Linked to Gene Expression Changes in Early Development

During early embryonic development, DNA methylation levels undergo significant changes (reviewed by Yang et al. 2007).

**Fig. 6:**
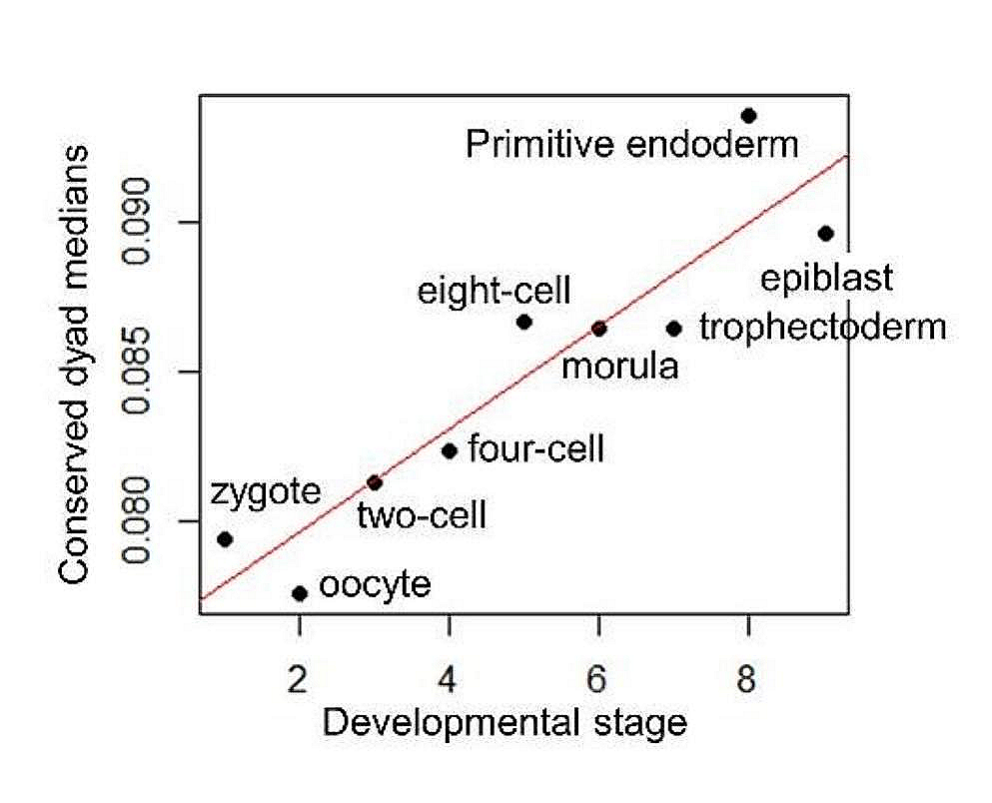
Density of conserved CpG codon dyads in expressed genes across human embryonic developmental stages. The median of the number of conserved CpG codon dyads (normalized by CDS length) is plotted against nine embryonic developmental stages. The red line represents a linear fit (*p* < 0.001, R^2^ = 0.85)

Leveraging publicly available expression data (Yan et al. 2013), we conducted tests to investigate the role of CpG codon dyads in human embryonic development. The density of conserved CpG codon dyads was assessed in the CDS of expressed genes (higher than 1 RPKM) at various stages of human embryonic development (Fig. 6). A linear model, using the median values, indicates a significant increase in the density of conserved CpG codon dyads as development progresses (F¬1,7 = 39.94, *p* < 0.001).

We carried out a similar analysis in mouse embryonic cells at different developmental stages using a dataset by Tang et al. (2011). Here too, the density of conserved CpG codon dyads exhibited a significant increase as development progressed, albeit with a smaller effect (*p* = 0.033, R2 = 0.48), possibly due to the lower number of CpG codon dyads present in mice (Fig. S7).

## Discussion

In this study, we present evidence of selection that either promotes or inhibits the presence of CpG codon dyads, which can be targeted for DNA methylation. We have discovered that the HOX gene group appears to exhibit an enrichment of CpG codon dyads, a result consistent with previous studies on codon methylation (Branciamore et al. 2010). Additionally, our findings suggest an over-representation of the RNA polymerase II transcription factor group in genes rich in CpG codon dyads. Conversely, gene groups associated with DNA damage repair and mitosis showed a lack or scarcity of CpG codon dyads.

These enriched gene sets provide valuable insights into the potential functional role of CpG codon dyads, as well as single codon CpGs. Given the well-established function of DNA methylation in regulating gene expression (Jin et al., 2011; Moore et al., 2013), it is plausible that it plays a similar role in this context. One possible mechanism through which this regulation could occur is gene silencing. HOX genes, for instance, are typically expressed only during development and are suppressed in most somatic cells—a process that may be mediated by the DNA methylation discussed here. This selective expression pattern stands in contrast to gene groups with very few CpG codon dyad sites. In normal circumstances, genes related to DNA damage repair and cell division would not be expected to be silenced, and as a result, they typically exhibit few or no CpG codon dyads. Supporting this notion, a previous study found that DNA damage repair genes are repressed through hypermethylation in cancerous cells (Catteau & Morris, 2002).

An alternative determinant that impact CpG codon dyads arises from the concept of codon usage bias (CUB), which has been extensively studied in the context of gene expression regulation (Zahdeh and Carmel 2019).

Zahdeh and Carmel (2019) demonstrated a strong preference for certain codons near the 3’ end of genes, implicating CUB in shaping the distribution of codons within coding sequences. While our observation of CpG codon dyads predominantly near the start of genes aligns with this notion, the widespread presence of these dyads throughout the coding sequence suggests a more complex interplay between CUB and other regulatory mechanisms. This is further supported by the dynamic expression trends observed throughout development, indicating that factors beyond CUB alone likely influence the distribution of CpG codon dyads.

The significance of CpG codon dyads at the beginning of coding sequences raises intriguing questions regarding their potential role in translation initiation, mRNA folding, and other processes associated with early stages of protein synthesis. While CUB may exert selective pressure on codon usage preferences, the observed enrichment of CpG codon dyads suggests additional layers of regulation that warrant further investigation. Future studies could explore the mechanistic basis of this phenomenon, perhaps through experimental validation of the proposed regulatory mechanisms or computational modeling to dissect the interplay between CUB, DNA methylation, and gene expression dynamics.

Our data adds a new layer of evidence that challenges the outdated view that synonymous codon variation represents neutral evolution. The phenomenon of CUB suggests that not all synonymous codons are equivalent and that they can have significant impacts on translation efficiency, mRNA stability, and protein folding (Plotkin and Kudla 2011; Sun and Zhang 2022). Our analysis suggests that a complete understanding of CUB should also consider the identity of consecutive codons (dyads) and their influence on CpG formation and DNA methylation.

Our analysis reveals a large number of conserved CpG dyads in mammalian genes, although many consist of a single site in the coding sequence (CDS). While the impact of a single conserved CpG might appear negligible, there are numerous examples of functional single CpG sites. For instance, a single CpG site is part of the Pax6 binding motif in the promoter of the Steroidogenic Acute Regulatory Protein (StAR), affecting its expression (Wang et al. 2011). In the peroxisomal membrane protein 24 (PMP24), hypermethylation of a single intronic CpG causes loss of mRNA expression (Zhang et al. 2010). Similarly, demethylation of a single CpG in the promoter region of EBPδ, a CCAAT/enhancer-binding protein transcription factor, was a determinant for enhanced expression (Ceccarelli et al. 2011). Additionally, hypomethylation of a single CpG in exon 2 of the cytokine IL-6 promotes expression, while deletion of another single site leads to reduced expression (Shi et al. 2022).

Our analysis unveiled a striking increase in the density of conserved CpG codon dyads throughout the course of embryonic development, as depicted in Fig. 6. This trend aligns closely with the dynamic alterations in DNA methylation that occur during development. In mice, a global DNA demethylation event occurs during the early stages of development, followed by subsequent DNA methylation during later stages (reviewed by Yang et al. 2007). This observation strongly suggests a functional role for these conserved CpG codon dyads, particularly as DNA methylation assumes increasing importance during the later stages of development.

### Electronic Supplementary Material

Below is the link to the electronic supplementary material.

Supplementary Material 1

## Data Availability

Code is available at GitHub (https://github.com/erantauber/AMCoR). This contains different variants of the code for different targets, as well as smaller sets of codes for specific analysis. Full description is therein.

## Data Availability

Eutherian coding sequence data is available at 10.5061/dryad.5dv41nsbc (Bowman et al. 2023). Human embryo expression data is available at 10.1038/nsmb.2660 (Yan et al. 2013). Mouse embryo expression data is available at 10.1371/journal.pone.0021208. (Tang et al. 2011). Bisulfite sequencing data was acquired from the Enocde’s Epigenomes from four individuals project (https://www.encodeproject.org/).
