# Supplementary material for: Interconnected Codons: Unravelling the Epigenetic Significance of Flanking Sequences in CpG Dyads
Source: J Mol Evol. 2024 Apr 18;92(3):207–16. doi: 10.1007/s00239-024-10172-1 (PMC11169003; doi:10.1007/s00239-024-10172-1)
Supplement: Supplementary file 1 — Supplementary Material 1 [file 239_2024_10172_MOESM1_ESM.docx]

Supplemental figures for:

**Interconnected Codons: Unravelling the Epigenetic Significance of Flanking Sequences in CpG Dyads**

Leo Douglas Creasey and Eran Tauber*

Department of Evolutionary and Environmental Biology, and Institute of Evolution, University of Haifa 199 Abba-Hushi Avenue, Haifa 3498838 Israel,

*To whom correspondence should be addressed.

[etauber@univ.haifa.ac.il](mailto:etauber@univ.haifa.ac.il)


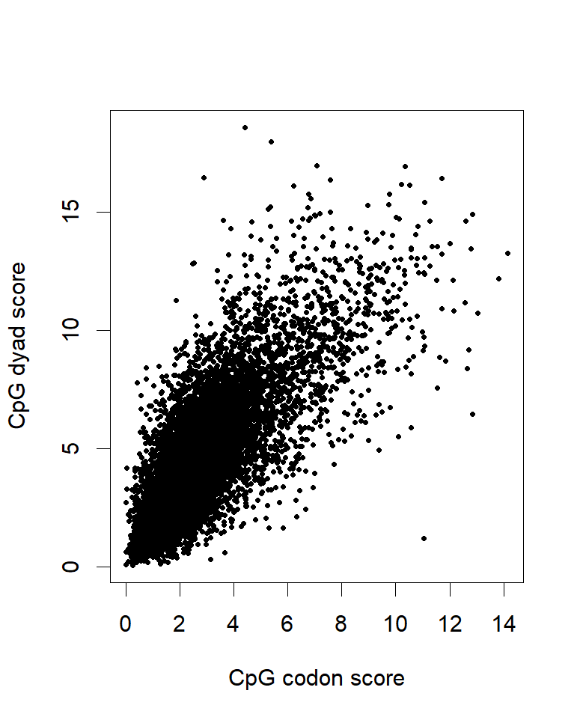


**Figure S1.** Correlation between CpG codon dyad density and single codon CpG frequency (NGC). Spearman correlation analysis across 13,491 eutherian mammalian genes demonstrates a strong positive correlation (ρ = 0.83, S = 6.7e+10, p < 2.2e-16) between the density of CpG codon dyads and the frequency of single codon CpG. A single outlier (*BCL6-AS1*) with an excessive dyad score has been excluded from the analysis.


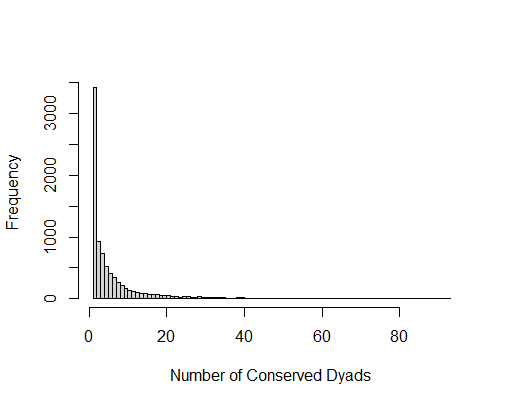


**Figure S2.** Distribution of conserved CpG codon dyads in mammalian genes. Conserved dyads are defined as present in over 75% of the species. The data consisted of 13,491 gene alignments of 261 eutherian mammal species.

**
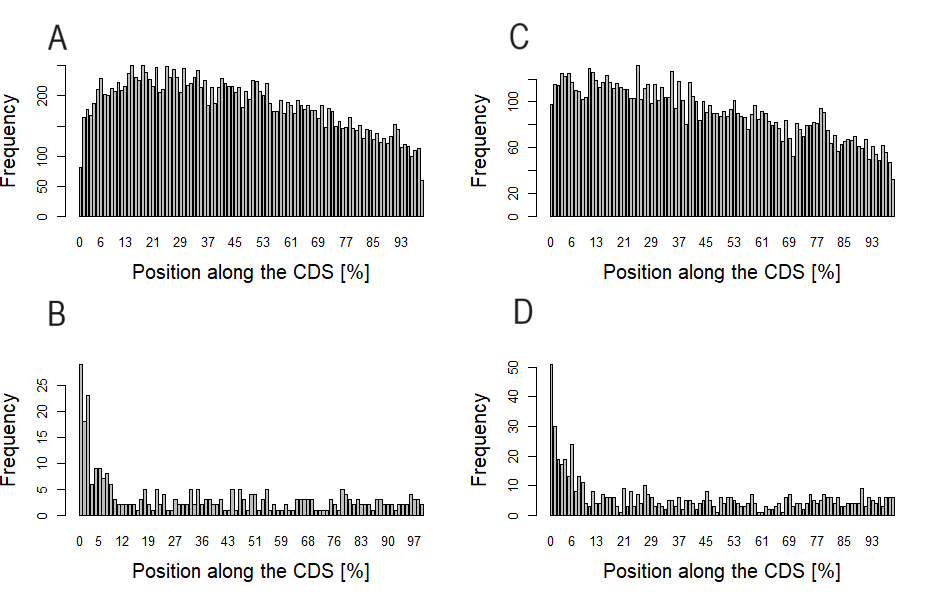
**

**Figure S3.** Distribution of conserved CpG sites along the Coding DNA Sequences (CDS). The histograms on the left (**A**, **B**) depict the distribution of CpG codon dyads (NNC-GCC). Panel **A** displays results from the top 1000 genes with the highest CpG codon dyad frequency, while Panel **B** shows the corresponding distribution for the lowest 1000 genes (with CpG number ≥ 1). Panels **C** and **D** illustrate the distribution of NCG codons within the top 1000 genes with the highest frequency and the lowest 1000 genes, respectively.


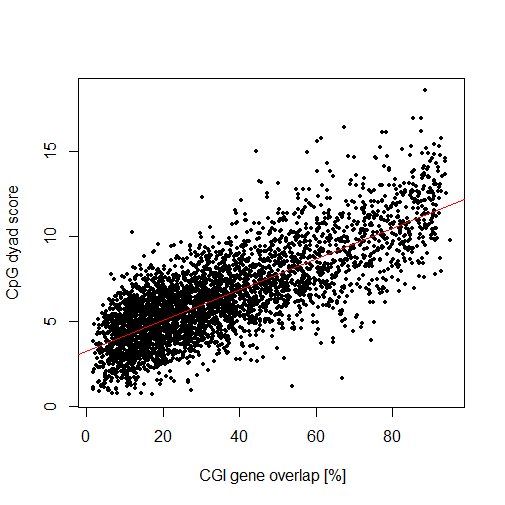


**Figure S4.** Correlation between the density of CpG codon dyads and CpG island (CGI) gene coverage in 13,491 human genes. Spearman correlation analysis demonstrates a positive correlation (ρ = 0.73, S = 2.5e+10, p < 2.2e-16) between the density of CpG codon dyads and the frequency of single codon CpG. The linear curve fit is also depicted (red).


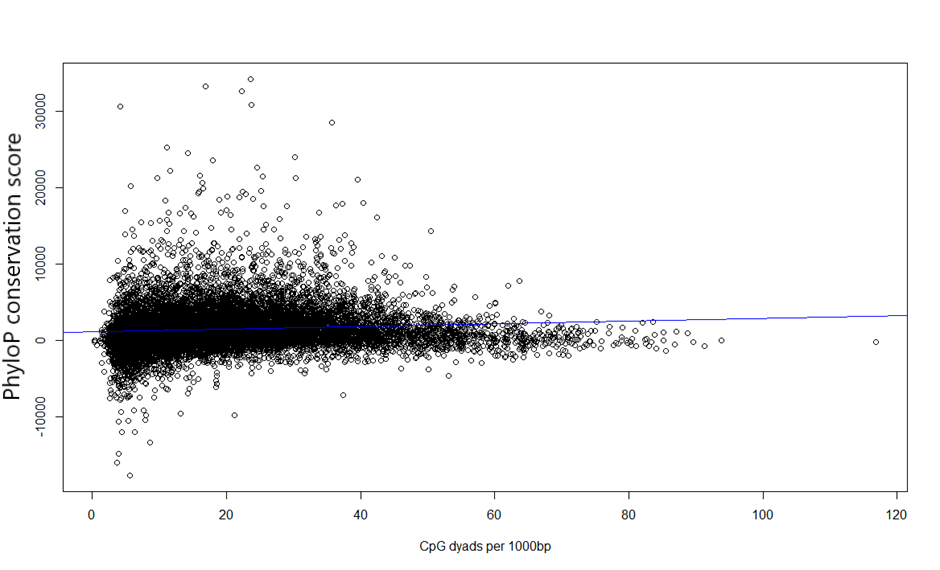


**Figure S5.** CpG codon dyad density exhibits a weak correlation with gene conservation scores. PhyloP conservation scores are plotted against the number of CpG codon dyads per 1000 bp. Positive numbers on the Y-axis indicate a gene is conserved, negative numbers imply that the gene is under accelerating evolution. The blue line represents a linear model curve fit (p< 2.2e-16, r^2^ = 0.007).


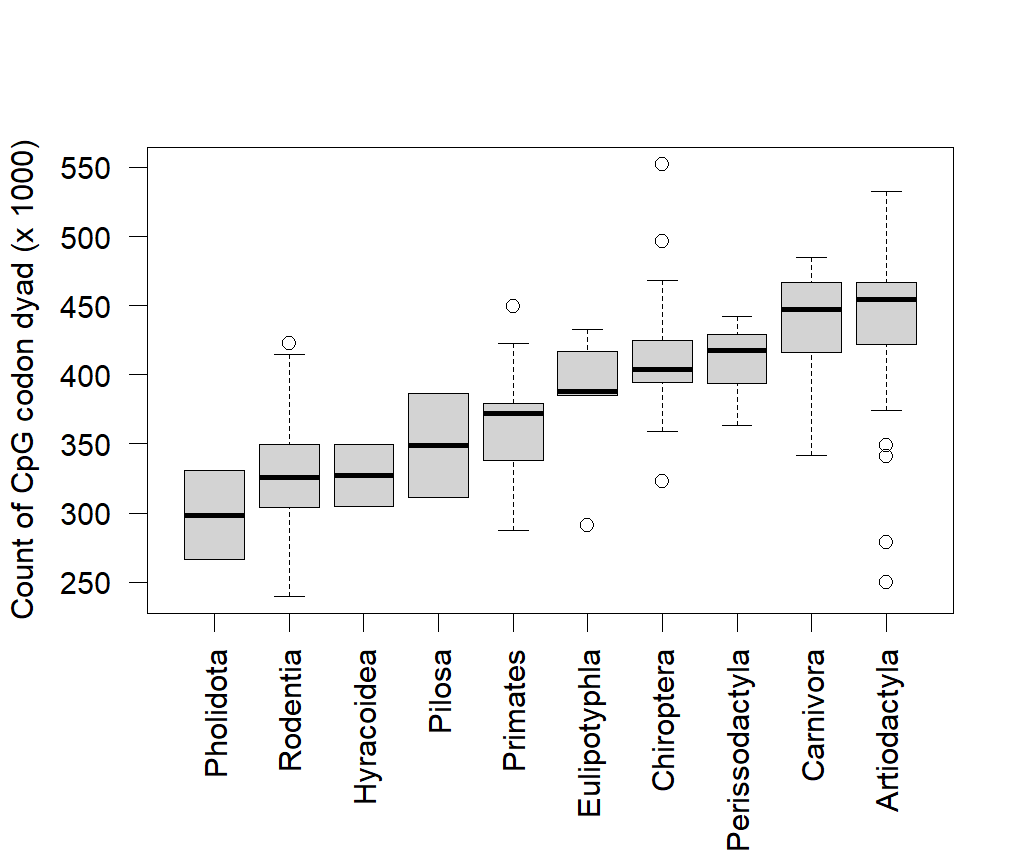


**Figure S6. CpG codon dyad counts across** **eutherian mammal orders.** Boxplot indicating the first and third quartiles, and the median (line within the box) of CpG codon dyad counts per genome. Orders that include less than three species were omitted. The difference between the orders is significant (Kruskal-Wallis test, χ^2^= 159.08, df = 9, p-value < 2.2e-16).


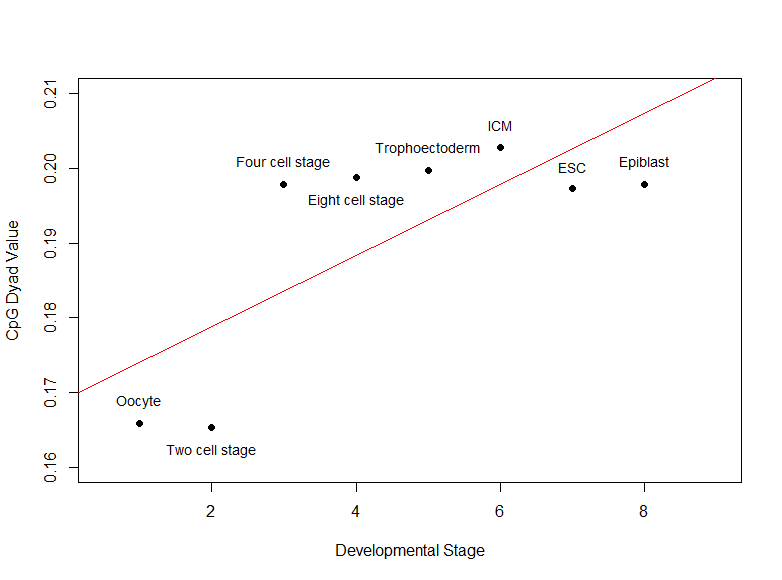


**Figure S7**. Median conserved CpG codon dyad value of genes expressed at the different stages of mice development, with a linear model overlaid (p= 0.033, R^2^ = 0.48). Transcriptional expression data are from Tang et al. 2011. *PLoS One*, **6**, e21208.
